# 4-Phenyl-9,12,15-trioxa-1,5,6,18-tetra­azatetra­cyclo­[16.6.1.0^2,6^.0^19,24^]penta­conta-2,4,19,21,23-pentaen-25-one

**DOI:** 10.1107/S1600536809037131

**Published:** 2009-09-19

**Authors:** Joseph Nathan Ghomsi, Noureddine Hamou Ahabchane, Rachid Bouhfid, El Mokhtar Essassi, Seik Weng Ng

**Affiliations:** a Laboratoire de Chimie Organique Hétérocyclique, Pôle de Compétences Pharmacochimie, Université Mohammed V-Agdal, BP 1014 Avenue Ibn Batout, Rabat, Morocco; b Institute of Nanomaterials and Nanotechnology, Avenue de l’Armée Royale, Madinat El Irfane, 10100 Rabat, Morocco; c Department of Chemistry, University of Malaya, 50603 Kuala Lumpur, Malaysia

## Abstract

The title compound, C_24_H_26_N_4_O_4_, is a diaza-crown ether encompassing linked phenylpyrazolyl and benzimidazole units that contribute five atoms to the 16-atom ring. The two planar phenylpyrazolyl and benzimidazole units are aligned at an angle of 66.4 (1)°. The carbonyl O atom of the benzimidazole unit is directed away from the middle of the ring.

## Related literature

For the selective recognition of sodium and potassium cyanide by diaza-crown ethers, see: Liu *et al.* (2005 ▶).
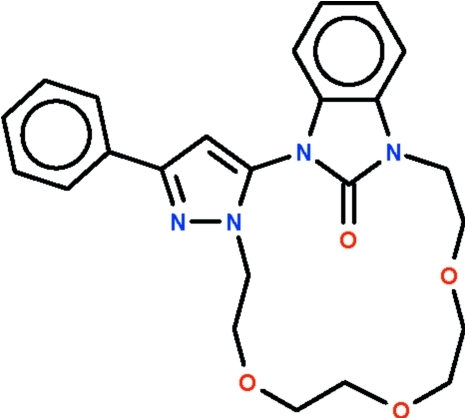


## Experimental

### 

#### Crystal data

C_24_H_26_N_4_O_4_
*M* *_r_* = 434.49
Monoclinic, 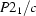

*a* = 13.348 (1) Å
*b* = 15.785 (1) Å
*c* = 11.007 (1) Å
β = 102.681 (1)°
*V* = 2262.6 (3) Å^3^
*Z* = 4
Mo *K*α radiation
μ = 0.09 mm^−1^
*T* = 293 K
0.30 × 0.30 × 0.20 mm

#### Data collection

Nonius KappaCCD diffractometer
Absorption correction: none
4728 measured reflections
4559 independent reflections
3705 reflections with *I* > 2σ(*I*)
*R*_int_ = 0.038

#### Refinement

*R*[*F* ^2^ > 2σ(*F* ^2^)] = 0.055
*wR*(*F* ^2^) = 0.176
*S* = 1.16
4559 reflections
289 parameters
H-atom parameters constrained
Δρ_max_ = 0.34 e Å^−3^
Δρ_min_ = −0.30 e Å^−3^

### 

Data collection: *KappaCCD Server Software* (Nonius, 1998 ▶); cell refinement: *KappaCCD Server Software*; data reduction: *DENZO* and *SCALEPACK* (Otwinowski & Minor, 1997 ▶); program(s) used to solve structure: *SHELXS97* (Sheldrick, 2008 ▶); program(s) used to refine structure: *SHELXL97* (Sheldrick, 2008 ▶); molecular graphics: *X-SEED* (Barbour, 2001 ▶); software used to prepare material for publication: *publCIF* (Westrip, 2009 ▶).

## Supplementary Material

Crystal structure: contains datablocks global, I. DOI: 10.1107/S1600536809037131/tk2541sup1.cif

Structure factors: contains datablocks I. DOI: 10.1107/S1600536809037131/tk2541Isup2.hkl

Additional supplementary materials:  crystallographic information; 3D view; checkCIF report

## Figures and Tables

**Fig. 1.**
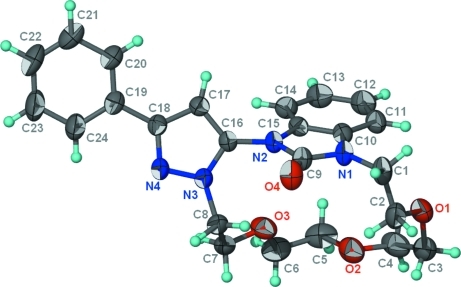
Thermal ellipsoid plot (Barbour, 2001) of C24H26N4O4 at the 50% probability level; hydrogen atoms are drawn as spheres of arbitrary radius.

## supplementary crystallographic information

### Experimental

1-(3-Phenylpyrazolyl)benzimidazol-2-one (1 g, 4.5 mmol), dichlorotetraethyleneglycol (0.31 ml, 5.0 mmol), potassium carbonate (0.7 g, 5.0 mmol) and a catalytic quantity of tetra-*n*-butylammonium bromide were stirred in *N*,*N*-dimethylformamide (60 ml) in an oil bath heated to 353–363 K for 24 h. The insoluble salts were filtered off and the solvent removed in vacuum. The residue was separated by chromatography on silica gel with a hexane:ethyl acetate (7:3) solvent system. Evaporation of the solvent gave the compound as colorless crystals in 25% yield; m.p. 402–404 K.

### Refinement

Carbon-bound H-atoms were placed in calculated positions (C—H 0.93 to 0.97 Å) and were included in the refinement in the riding model approximation, with *U*(H) set to 1.2*U*(C).

### Crystal data

**Table:**

C_24_H_26_N_4_O_4_	*F*(000) = 920
*M**_r_* = 434.49	*D*_x_ = 1.276 Mg m^−^^3^
Monoclinic, *P*2_1_/*c*	Mo *K*α radiation, λ = 0.71073 Å
Hall symbol: -P 2ybc	Cell parameters from 4728 reflections
*a* = 13.348 (1) Å	θ = 1.3–26.4°
*b* = 15.785 (1) Å	µ = 0.09 mm^−^^1^
*c* = 11.007 (1) Å	*T* = 293 K
β = 102.681 (1)°	Prism, colorless
*V* = 2262.6 (3) Å^3^	0.30 × 0.30 × 0.20 mm
*Z* = 4	

### Data collection

**Table:**

Nonius KappaCCD diffractometer	3705 reflections with *I* > 2σ(*I*)
Radiation source: fine-focus sealed tube	*R*_int_ = 0.038
graphite	θ_max_ = 26.4°, θ_min_ = 1.6°
φ scans	*h* = −16→16
4728 measured reflections	*k* = 0→19
4559 independent reflections	*l* = 0→13

### Refinement

**Table:**

Refinement on *F*^2^	Primary atom site location: structure-invariant direct methods
Least-squares matrix: full	Secondary atom site location: difference Fourier map
*R*[*F*^2^ > 2σ(*F*^2^)] = 0.055	Hydrogen site location: inferred from neighbouring sites
*wR*(*F*^2^) = 0.176	H-atom parameters constrained
*S* = 1.16	*w* = 1/[σ^2^(*F*_o_^2^) + (0.0966*P*)^2^ + 0.3451*P*] where *P* = (*F*_o_^2^ + 2*F*_c_^2^)/3
4559 reflections	(Δ/σ)_max_ < 0.001
289 parameters	Δρ_max_ = 0.34 e Å^−^^3^
0 restraints	Δρ_min_ = −0.30 e Å^−^^3^

### Fractional atomic coordinates and isotropic or equivalent isotropic displacement parameters (Å^2^)

**Table:**

	*x*	*y*	*z*	*U*_iso_*/*U*_eq_	
O1	0.87698 (11)	0.57891 (11)	0.53630 (16)	0.0794 (5)	
O2	0.74157 (12)	0.43571 (10)	0.44390 (16)	0.0745 (4)	
O3	0.53984 (12)	0.47052 (9)	0.31375 (15)	0.0699 (4)	
O4	0.54645 (11)	0.71208 (10)	0.41785 (13)	0.0682 (4)	
N1	0.70082 (11)	0.67744 (10)	0.36647 (14)	0.0524 (4)	
N2	0.55695 (10)	0.65098 (9)	0.22803 (13)	0.0449 (3)	
N3	0.38396 (11)	0.60439 (10)	0.22449 (13)	0.0499 (4)	
N4	0.28762 (10)	0.61095 (10)	0.15421 (14)	0.0520 (4)	
C1	0.76670 (16)	0.69810 (14)	0.4862 (2)	0.0661 (5)	
H1A	0.7322	0.7392	0.5282	0.079*	
H1B	0.8293	0.7240	0.4728	0.079*	
C2	0.79396 (16)	0.62171 (18)	0.5685 (2)	0.0753 (6)	
H2A	0.8125	0.6392	0.6550	0.090*	
H2B	0.7352	0.5841	0.5583	0.090*	
C3	0.88970 (18)	0.4930 (2)	0.5761 (3)	0.0900 (8)	
H3A	0.8534	0.4834	0.6422	0.108*	
H3B	0.9620	0.4816	0.6091	0.108*	
C4	0.84994 (19)	0.43449 (18)	0.4718 (3)	0.0905 (9)	
H4A	0.8749	0.4516	0.3991	0.109*	
H4B	0.8743	0.3775	0.4943	0.109*	
C5	0.6978 (2)	0.39909 (15)	0.3267 (2)	0.0809 (7)	
H5A	0.7286	0.3441	0.3198	0.097*	
H5B	0.7112	0.4350	0.2605	0.097*	
C6	0.5855 (2)	0.38942 (13)	0.3138 (2)	0.0767 (6)	
H6A	0.5561	0.3602	0.2367	0.092*	
H6B	0.5720	0.3560	0.3824	0.092*	
C7	0.43698 (16)	0.46653 (14)	0.32369 (19)	0.0647 (5)	
H7A	0.4311	0.4322	0.3949	0.078*	
H7B	0.3954	0.4409	0.2494	0.078*	
C8	0.40064 (15)	0.55449 (14)	0.33892 (17)	0.0605 (5)	
H8A	0.3369	0.5517	0.3673	0.073*	
H8B	0.4509	0.5831	0.4027	0.073*	
C9	0.59594 (13)	0.68361 (11)	0.34668 (17)	0.0499 (4)	
C10	0.72822 (13)	0.64095 (10)	0.26302 (17)	0.0471 (4)	
C11	0.82330 (14)	0.62168 (12)	0.2396 (2)	0.0590 (5)	
H11	0.8837	0.6329	0.2978	0.071*	
C12	0.82438 (17)	0.58505 (14)	0.1261 (2)	0.0685 (6)	
H12	0.8871	0.5717	0.1074	0.082*	
C13	0.73509 (17)	0.56767 (14)	0.0395 (2)	0.0652 (5)	
H13	0.7389	0.5424	−0.0356	0.078*	
C14	0.63960 (15)	0.58741 (12)	0.06293 (17)	0.0547 (4)	
H14	0.5792	0.5761	0.0047	0.066*	
C15	0.63804 (12)	0.62421 (10)	0.17555 (16)	0.0441 (4)	
C16	0.45233 (12)	0.64657 (10)	0.17250 (15)	0.0438 (4)	
C17	0.39923 (13)	0.68079 (10)	0.06243 (16)	0.0461 (4)	
H17	0.4253	0.7126	0.0053	0.055*	
C18	0.29661 (13)	0.65690 (10)	0.05540 (15)	0.0460 (4)	
C19	0.20534 (13)	0.67861 (11)	−0.04115 (16)	0.0508 (4)	
C20	0.20897 (16)	0.74172 (13)	−0.12711 (18)	0.0602 (5)	
H20	0.2698	0.7715	−0.1231	0.072*	
C21	0.12347 (19)	0.76134 (16)	−0.2191 (2)	0.0749 (6)	
H21	0.1273	0.8037	−0.2766	0.090*	
C22	0.0333 (2)	0.71854 (18)	−0.2258 (2)	0.0863 (8)	
H22	−0.0239	0.7314	−0.2882	0.104*	
C23	0.02757 (19)	0.65628 (19)	−0.1396 (3)	0.0910 (8)	
H23	−0.0339	0.6277	−0.1434	0.109*	
C24	0.11280 (16)	0.63617 (15)	−0.0476 (2)	0.0723 (6)	
H24	0.1083	0.5942	0.0102	0.087*	

### Atomic displacement parameters (Å^2^)

**Table:**

	*U*^11^	*U*^22^	*U*^33^	*U*^12^	*U*^13^	*U*^23^
O1	0.0499 (8)	0.0938 (11)	0.0948 (11)	−0.0035 (7)	0.0166 (7)	0.0240 (9)
O2	0.0620 (9)	0.0817 (10)	0.0879 (11)	0.0055 (7)	0.0339 (8)	0.0023 (8)
O3	0.0719 (10)	0.0537 (8)	0.0884 (11)	0.0005 (6)	0.0270 (8)	0.0132 (7)
O4	0.0574 (8)	0.0837 (10)	0.0644 (8)	0.0036 (7)	0.0153 (6)	−0.0271 (7)
N1	0.0391 (8)	0.0581 (9)	0.0564 (9)	−0.0015 (6)	0.0025 (6)	−0.0121 (7)
N2	0.0364 (7)	0.0515 (8)	0.0457 (7)	−0.0022 (5)	0.0066 (5)	−0.0064 (6)
N3	0.0381 (7)	0.0627 (9)	0.0476 (8)	−0.0022 (6)	0.0067 (6)	0.0078 (6)
N4	0.0375 (7)	0.0633 (9)	0.0525 (8)	−0.0039 (6)	0.0037 (6)	0.0032 (7)
C1	0.0534 (11)	0.0746 (13)	0.0643 (12)	−0.0117 (9)	−0.0002 (9)	−0.0193 (10)
C2	0.0507 (11)	0.1098 (18)	0.0612 (12)	−0.0070 (11)	0.0032 (9)	0.0040 (12)
C3	0.0520 (12)	0.110 (2)	0.106 (2)	0.0051 (13)	0.0119 (12)	0.0449 (17)
C4	0.0650 (14)	0.0863 (17)	0.133 (2)	0.0198 (12)	0.0494 (15)	0.0357 (17)
C5	0.105 (2)	0.0658 (13)	0.0814 (16)	0.0234 (12)	0.0423 (14)	0.0053 (12)
C6	0.1036 (19)	0.0526 (11)	0.0731 (14)	0.0036 (11)	0.0177 (13)	−0.0005 (10)
C7	0.0582 (12)	0.0722 (13)	0.0589 (11)	−0.0143 (9)	0.0027 (9)	0.0201 (10)
C8	0.0509 (10)	0.0840 (14)	0.0463 (9)	−0.0043 (9)	0.0101 (8)	0.0145 (9)
C9	0.0461 (9)	0.0509 (9)	0.0516 (9)	0.0000 (7)	0.0082 (7)	−0.0095 (8)
C10	0.0423 (9)	0.0435 (8)	0.0556 (10)	−0.0006 (6)	0.0107 (7)	0.0002 (7)
C11	0.0424 (9)	0.0630 (11)	0.0724 (12)	0.0028 (8)	0.0143 (8)	0.0057 (9)
C12	0.0590 (12)	0.0738 (13)	0.0806 (14)	0.0150 (10)	0.0328 (11)	0.0071 (11)
C13	0.0731 (13)	0.0671 (12)	0.0615 (11)	0.0087 (10)	0.0280 (10)	−0.0040 (10)
C14	0.0588 (11)	0.0551 (10)	0.0507 (9)	−0.0019 (8)	0.0133 (8)	−0.0033 (8)
C15	0.0431 (9)	0.0399 (8)	0.0509 (9)	−0.0011 (6)	0.0140 (7)	−0.0008 (7)
C16	0.0385 (8)	0.0468 (8)	0.0457 (8)	−0.0022 (6)	0.0083 (6)	−0.0028 (7)
C17	0.0469 (9)	0.0439 (8)	0.0473 (9)	−0.0019 (7)	0.0099 (7)	0.0013 (7)
C18	0.0443 (9)	0.0443 (8)	0.0469 (9)	−0.0001 (6)	0.0046 (7)	−0.0029 (7)
C19	0.0495 (10)	0.0497 (9)	0.0496 (9)	0.0060 (7)	0.0030 (7)	−0.0080 (8)
C20	0.0595 (11)	0.0609 (11)	0.0555 (10)	0.0093 (9)	0.0029 (8)	0.0008 (9)
C21	0.0790 (15)	0.0770 (14)	0.0611 (12)	0.0232 (12)	−0.0009 (10)	0.0070 (10)
C22	0.0712 (15)	0.0955 (17)	0.0761 (15)	0.0222 (13)	−0.0190 (12)	−0.0039 (13)
C23	0.0541 (13)	0.1033 (19)	0.0994 (19)	−0.0065 (12)	−0.0181 (12)	0.0013 (16)
C24	0.0528 (11)	0.0753 (14)	0.0789 (14)	−0.0048 (10)	−0.0071 (10)	0.0058 (11)

### Geometric parameters (Å, °)

**Table:**

O1—C2	1.408 (3)	C6—H6B	0.9700
O1—C3	1.424 (3)	C7—C8	1.492 (3)
O2—C4	1.411 (3)	C7—H7A	0.9700
O2—C5	1.418 (3)	C7—H7B	0.9700
O3—C7	1.402 (2)	C8—H8A	0.9700
O3—C6	1.418 (3)	C8—H8B	0.9700
O4—C9	1.216 (2)	C10—C11	1.383 (2)
N1—C9	1.372 (2)	C10—C15	1.392 (2)
N1—C10	1.395 (2)	C11—C12	1.380 (3)
N1—C1	1.452 (2)	C11—H11	0.9300
N2—C9	1.394 (2)	C12—C13	1.380 (3)
N2—C16	1.398 (2)	C12—H12	0.9300
N2—C15	1.399 (2)	C13—C14	1.390 (3)
N3—N4	1.3513 (19)	C13—H13	0.9300
N3—C16	1.354 (2)	C14—C15	1.373 (3)
N3—C8	1.460 (2)	C14—H14	0.9300
N4—C18	1.334 (2)	C16—C17	1.373 (2)
C1—C2	1.504 (3)	C17—C18	1.406 (2)
C1—H1A	0.9700	C17—H17	0.9300
C1—H1B	0.9700	C18—C19	1.471 (2)
C2—H2A	0.9700	C19—C20	1.382 (3)
C2—H2B	0.9700	C19—C24	1.393 (3)
C3—C4	1.478 (4)	C20—C21	1.385 (3)
C3—H3A	0.9700	C20—H20	0.9300
C3—H3B	0.9700	C21—C22	1.368 (4)
C4—H4A	0.9700	C21—H21	0.9300
C4—H4B	0.9700	C22—C23	1.380 (4)
C5—C6	1.482 (4)	C22—H22	0.9300
C5—H5A	0.9700	C23—C24	1.384 (3)
C5—H5B	0.9700	C23—H23	0.9300
C6—H6A	0.9700	C24—H24	0.9300
			
C2—O1—C3	115.28 (18)	N3—C8—C7	113.37 (17)
C4—O2—C5	113.0 (2)	N3—C8—H8A	108.9
C7—O3—C6	112.79 (17)	C7—C8—H8A	108.9
C9—N1—C10	109.93 (14)	N3—C8—H8B	108.9
C9—N1—C1	121.44 (16)	C7—C8—H8B	108.9
C10—N1—C1	128.23 (16)	H8A—C8—H8B	107.7
C9—N2—C16	124.04 (14)	O4—C9—N1	127.12 (17)
C9—N2—C15	109.56 (13)	O4—C9—N2	126.59 (16)
C16—N2—C15	126.40 (14)	N1—C9—N2	106.29 (14)
N4—N3—C16	111.43 (13)	C11—C10—C15	121.28 (17)
N4—N3—C8	118.73 (14)	C11—C10—N1	131.19 (17)
C16—N3—C8	129.83 (15)	C15—C10—N1	107.53 (14)
C18—N4—N3	105.29 (13)	C12—C11—C10	116.95 (19)
N1—C1—C2	112.63 (18)	C12—C11—H11	121.5
N1—C1—H1A	109.1	C10—C11—H11	121.5
C2—C1—H1A	109.1	C11—C12—C13	121.98 (18)
N1—C1—H1B	109.1	C11—C12—H12	119.0
C2—C1—H1B	109.1	C13—C12—H12	119.0
H1A—C1—H1B	107.8	C12—C13—C14	121.01 (19)
O1—C2—C1	109.45 (19)	C12—C13—H13	119.5
O1—C2—H2A	109.8	C14—C13—H13	119.5
C1—C2—H2A	109.8	C15—C14—C13	117.28 (18)
O1—C2—H2B	109.8	C15—C14—H14	121.4
C1—C2—H2B	109.8	C13—C14—H14	121.4
H2A—C2—H2B	108.2	C14—C15—C10	121.50 (16)
O1—C3—C4	110.9 (2)	C14—C15—N2	131.82 (16)
O1—C3—H3A	109.4	C10—C15—N2	106.68 (14)
C4—C3—H3A	109.4	N3—C16—C17	107.53 (14)
O1—C3—H3B	109.4	N3—C16—N2	122.76 (15)
C4—C3—H3B	109.5	C17—C16—N2	129.72 (15)
H3A—C3—H3B	108.0	C16—C17—C18	104.54 (15)
O2—C4—C3	109.7 (2)	C16—C17—H17	127.7
O2—C4—H4A	109.7	C18—C17—H17	127.7
C3—C4—H4A	109.7	N4—C18—C17	111.20 (15)
O2—C4—H4B	109.7	N4—C18—C19	120.47 (16)
C3—C4—H4B	109.7	C17—C18—C19	128.30 (17)
H4A—C4—H4B	108.2	C20—C19—C24	118.42 (18)
O2—C5—C6	109.61 (18)	C20—C19—C18	120.97 (17)
O2—C5—H5A	109.7	C24—C19—C18	120.61 (18)
C6—C5—H5A	109.7	C19—C20—C21	121.0 (2)
O2—C5—H5B	109.7	C19—C20—H20	119.5
C6—C5—H5B	109.7	C21—C20—H20	119.5
H5A—C5—H5B	108.2	C22—C21—C20	120.2 (2)
O3—C6—C5	109.42 (19)	C22—C21—H21	119.9
O3—C6—H6A	109.8	C20—C21—H21	119.9
C5—C6—H6A	109.8	C21—C22—C23	119.7 (2)
O3—C6—H6B	109.8	C21—C22—H22	120.1
C5—C6—H6B	109.8	C23—C22—H22	120.1
H6A—C6—H6B	108.2	C22—C23—C24	120.4 (2)
O3—C7—C8	108.36 (16)	C22—C23—H23	119.8
O3—C7—H7A	110.0	C24—C23—H23	119.8
C8—C7—H7A	110.0	C23—C24—C19	120.3 (2)
O3—C7—H7B	110.0	C23—C24—H24	119.9
C8—C7—H7B	110.0	C19—C24—H24	119.9
H7A—C7—H7B	108.4		
			
C16—N3—N4—C18	1.09 (19)	C11—C10—C15—C14	0.5 (3)
C8—N3—N4—C18	−177.89 (16)	N1—C10—C15—C14	−179.58 (16)
C9—N1—C1—C2	94.0 (2)	C11—C10—C15—N2	179.97 (16)
C10—N1—C1—C2	−78.1 (3)	N1—C10—C15—N2	−0.06 (18)
C3—O1—C2—C1	−160.4 (2)	C9—N2—C15—C14	179.20 (18)
N1—C1—C2—O1	82.7 (2)	C16—N2—C15—C14	−0.8 (3)
C2—O1—C3—C4	101.9 (2)	C9—N2—C15—C10	−0.24 (18)
C5—O2—C4—C3	164.8 (2)	C16—N2—C15—C10	179.74 (15)
O1—C3—C4—O2	−73.7 (2)	N4—N3—C16—C17	−1.25 (19)
C4—O2—C5—C6	170.39 (18)	C8—N3—C16—C17	177.59 (18)
C7—O3—C6—C5	−169.59 (19)	N4—N3—C16—N2	178.75 (15)
O2—C5—C6—O3	64.1 (2)	C8—N3—C16—N2	−2.4 (3)
C6—O3—C7—C8	172.95 (17)	C9—N2—C16—N3	−58.0 (2)
N4—N3—C8—C7	97.73 (19)	C15—N2—C16—N3	122.04 (19)
C16—N3—C8—C7	−81.0 (2)	C9—N2—C16—C17	122.0 (2)
O3—C7—C8—N3	73.3 (2)	C15—N2—C16—C17	−58.0 (3)
C10—N1—C9—O4	179.75 (19)	N3—C16—C17—C18	0.86 (18)
C1—N1—C9—O4	6.3 (3)	N2—C16—C17—C18	−179.14 (16)
C10—N1—C9—N2	−0.49 (19)	N3—N4—C18—C17	−0.51 (19)
C1—N1—C9—N2	−173.91 (16)	N3—N4—C18—C19	−178.65 (15)
C16—N2—C9—O4	0.2 (3)	C16—C17—C18—N4	−0.21 (19)
C15—N2—C9—O4	−179.79 (19)	C16—C17—C18—C19	177.74 (16)
C16—N2—C9—N1	−179.53 (15)	N4—C18—C19—C20	164.40 (17)
C15—N2—C9—N1	0.45 (19)	C17—C18—C19—C20	−13.4 (3)
C9—N1—C10—C11	−179.68 (18)	N4—C18—C19—C24	−15.2 (3)
C1—N1—C10—C11	−6.8 (3)	C17—C18—C19—C24	166.97 (19)
C9—N1—C10—C15	0.4 (2)	C24—C19—C20—C21	−1.3 (3)
C1—N1—C10—C15	173.20 (18)	C18—C19—C20—C21	179.05 (18)
C15—C10—C11—C12	−0.2 (3)	C19—C20—C21—C22	0.4 (3)
N1—C10—C11—C12	179.87 (19)	C20—C21—C22—C23	0.7 (4)
C10—C11—C12—C13	−0.4 (3)	C21—C22—C23—C24	−0.9 (4)
C11—C12—C13—C14	0.7 (3)	C22—C23—C24—C19	0.0 (4)
C12—C13—C14—C15	−0.4 (3)	C20—C19—C24—C23	1.1 (3)
C13—C14—C15—C10	−0.2 (3)	C18—C19—C24—C23	−179.2 (2)
C13—C14—C15—N2	−179.54 (18)		
